# Community-Based Adaptation and Evaluation of a Peer-Led Intervention to Address Alcohol Use and HIV in Pregnant and Breastfeeding Women in South Africa: Protocol for the “Mentor Mothers Plus” Randomized Control Trial

**DOI:** 10.2196/78856

**Published:** 2025-12-18

**Authors:** Zaynab Essack, Zaino Petersen, Amanda P Miller, Sarah Schoetz Dean, Danielle Daniels, Hayley Hofmeester, Thomas Belin, Heidi van Rooyen, Jaco Louw, Dvora Joseph Davey

**Affiliations:** 1 Human Sciences Research Council Pretoria South Africa; 2 University of KwaZulu-Natal Pietermaritzburg South Africa; 3 Department of Epidemiology and Biostatistics School of Public Health San Diego State University San Diego United States; 4 Department of Epidemiology Fielding School of Public Health University of California, Los Angeles Los Angeles, CA United States; 5 Foundation for Alcohol Related Research Cape Town South Africa; 6 Department of Biostatistics Fielding School of Public Health University of California, Los Angeles Los Angeles, CA United States; 7 Department of Global Health School of Public Health University of Washington Seattle, WA United States; 8 Division of Infectious Diseases David Geffen School of Medicine University of California, Los Angeles Los Angeles, CA United States

**Keywords:** alcohol, HIV, peer-led intervention, pregnant and lactating women, community-based approach

## Abstract

**Background:**

In South Africa, pregnant and lactating women (PLW) face a dual burden of high alcohol use and HIV prevalence, both of which adversely affect maternal and infant health. However, few interventions address alcohol use and HIV risk concurrently in this population.

**Objective:**

This study seeks to adapt and pilot-test in a randomized controlled trial (RCT) the peer-led multisession intervention Mentor Mothers Plus (MM+) to integrate alcohol reduction strategies with HIV prevention and care support (in a seroneutral intervention) among PLW in Saldanha Bay, Western Cape, South Africa.

**Methods:**

Using the ADAPT-ITT (assessment, decision, adaptation, production, topical experts, integration, training, testing) framework, we adapted the evidence-based Mentor Mother (MM) model, originally aimed at reducing vertical HIV transmission among mothers with HIV. Our mixed methods design includes (1) qualitative research, including in-depth interviews (IDIs) and focus group discussions (FGDs), to assess drivers of perinatal alcohol use and HIV risk; (2) intervention adaptation through community-based participatory workshops with stakeholders to refine the MM+ intervention in order to include culturally and contextually strategies; and (3) a pilot RCT with 100 pregnant women (≥16 years old, reporting recent alcohol use) randomized in a 1:1 ratio to the MM+ intervention (motivational interviewing [MI], including HIV and alcohol reduction messaging) or enhanced standard of care (SOC). Primary RCT outcomes include reduced alcohol use, via phosphatidylethanol (PEth) blood levels at 6-month follow-up. Secondary outcomes include uptake and adherence to pre-exposure prophylaxis (PrEP) and antiretroviral therapy (ART) among women with HIV, as well as intervention fidelity, feasibility, and acceptability.

**Results:**

Community research included 2 FGDs with health workers (n=6) and community leaders (n=7) and 31 interviews with partners and peers of PLW. Data showed that women are motivated to reduce alcohol use for child health but face barriers (eg, misconceptions about safety of alcohol use, poverty, trauma, and intimate partner violence [IPV]). Alcohol use is with friends/family members/partners and tied to financial stress, unplanned pregnancy, and poor mental health. MM+ support preferences emphasize nonjudgmental, peer-based mentorship, family involvement, and faith/community engagement. Gaps include stigma around HIV and contraceptive services. Communities recommend mentor mothers with lived experience, providing culturally relevant, empathetic support, and linking to social and clinical services. In August 2025, a community workshop refined the intervention, emphasizing the need for trustworthy mentor mothers with lived experience. Priorities included culturally relevant information on alcohol use, HIV prevention, family planning, trauma and interpersonal violence support, and flexible delivery. Next steps involve finalizing materials, training mentor mothers, and launching the RCT.

**Conclusions:**

Our community-based, participatory study, MM+, offers a promising approach to address co-occurring alcohol use and HIV among PLW. Stakeholder engagement enhances contextual relevance and supports future scalability. The RCT will evaluate efficacy of the model in reducing alcohol and improving HIV outcomes.

**Trial Registration:**

Clinicaltrials.gov NCT06962592; https://clinicaltrials.gov/study/NCT06962592

**International Registered Report Identifier (IRRID):**

DERR1-10.2196/78856

## Introduction

### Background

South Africa bears the highest global burden of both alcohol use during pregnancy and HIV prevalence. [1-3]. These interlinked public health crises are particularly concerning for pregnant and lactating women (PLW) due to their compounded impact on maternal, fetal, and child health. In the Western Cape province in South Africa, alcohol use during pregnancy is prevalent, contributing to some of the world’s highest rates of fetal alcohol spectrum disorder (FASD), with estimates ranging from 64 to 310 per 1000 live births [4,5]. Further, breastfeeding while consuming alcohol can impair child neurodevelopment, emphasizing the need for intervention throughout the perinatal period [6].

Pregnancy and postpartum are also periods of heightened risk for HIV, with the likelihood risk of acquisition more than doubling during this time [7]. Recent formative work among pregnant women at risk of HIV in Cape Town found that pregnant women who engage in alcohol use are more likely to report having recent condomless sex and multiple sex partners [8]. Qualitative work contextualized these findings, suggesting that alcohol use, HIV risk behaviors, nonadherence to HIV treatment, and experiences of intimate partner violence (IPV) are all inextricably linked health issues [9,10]. Despite the Joint United Nations Programme on HIV/AIDS (UNAIDS) and the World Health Organization’s (WHO) 2025 targets to eliminate vertical HIV transmission (defined as <2% HIV transmission or <50 cases per 100,000 live births) [11], South Africa’s pediatric HIV incidence still exceeds 750 per 100,000 live births. This is largely driven by a 30% antenatal HIV prevalence [11]. Considering the high HIV incidence in pregnancy, access to oral and other forms of pre-exposure prophylaxis (PrEP) are essential for PLW who do not have HIV [12,13], while women with HIV must be on and adhere to antiretroviral therapy (ART) to mitigate vertical HIV transmission [14-16]. However, alcohol use interferes with HIV care engagement, delaying ART initiation, reducing adherence, and ultimately increasing the risk of vertical HIV transmission [14-17]. Given the evidence that alcohol use disrupts biomedical HIV prevention and treatment, adherence-targeted interventions are critical.

Despite these risks, community-informed interventions targeting both HIV and alcohol use among PLW are lacking. The few existing brief interventions targeting alcohol use during pregnancy have shown limited effectiveness, in part due to short follow-up periods and the absence of biomarker validation [18,19]. Integrating peer mentor mothers into existing care structures is one promising evidence-based approach that has improved maternal health outcomes and reduced vertical HIV transmission in South Africa [20,21]. The existing Mentor Mother (MM) intervention, implemented by peer mothers to mothers, leverages task shifting, where positive deviant peer mothers provide counseling and support, typically through home-based delivery. The prior models have focused mostly on prevention of vertical HIV transmission but do not include any messaging on alcohol or substance use reduction. The previous interventions have been successfully adapted to address broader maternal-child health outcomes in Africa and have shown promise in reducing alcohol use [22]. However, alcohol has never been the primary focus of MM intervention content. Guided by ADAPT-ITT (assessment, decision, adaptation, production, topical experts, integration, training, testing), a framework developed to systematically adapt evidence-based HIV interventions, our study seeks to adapt the MM approach to reduce alcohol use and improve HIV prevention and treatment outcomes among PLW in South Africa [23].

Brief interventions for alcohol use in pregnancy have shown limited effectiveness. Although interventions such as the Philani Program Plus (an MM model) have shown reduction in alcohol use over extended periods, achieving early abstinence remains challenging [24]. Our study aims to bridge these gaps by integrating motivational interviewing (MI) techniques to assist with reduction in alcohol or other substance use through peers with lived experience of being pregnant and reducing or stopping alcohol use during pregnancy and breastfeeding.

The MM approach is well suited for this context, offering a cost-effective, accessible, and scalable approach through leveraging existing community health worker structures [25-27]. The model is highly adaptable, having been successfully implemented to address diverse health challenges across Africa, including HIV, nutrition, and child development [28,29]. The approach has also demonstrated effectiveness, producing significant reductions in vertical HIV transmission and improved ART adherence [27,30]. In addition to this approach, to further enhance the intervention impact, we will integrate MI—another evidence-based strategy shown to reduce alcohol use among PLW—into our adapted MM model [19,31].

### Theoretical Approach: Theory-of-Change Framework

Given the sociocultural complexities of alcohol use and HIV risk among PLW in South Africa, identifying a theory of change is critical to understanding the causal pathways that drive these behaviors and designing a responsive intervention [32]. The theory of change is an outcomes-based approach to understanding health behavior that maps how and why change occurs, articulating causal linkages between activities, outcomes, assumptions, and contextual factors [33].

The theory-of-change model identifies the multilevel determinants influencing alcohol use and HIV risk among PLW, including individual (eg, stress, depression), interpersonal (eg, intimate partner violence [IPV], peer influence), and community (eg, social norms, poverty) factors [34-36]. Key components of this framework include long-term (reduced maternal alcohol use, improved HIV prevention behaviors, and decreased vertical HIV transmission), intermediate (improved alcohol use disclosure, increased uptake and adherence to PrEP/ART, and reduced stigma in accessing care), and immediate (increased knowledge and motivation to reduce alcohol use, improved access to health services, and strengthened peer and community support systems) outcomes.

## Methods

### Study Design

This study was registered at ClinicalTrials.gov (NCT06962592, v1.0). The study is being implemented in Saldanha Bay, located in Western Cape, South Africa, which has a high burden of HIV and PLW who use alcohol. This periurban/rural fishing community has a population of over 150,000 people. The prevalence of FASD in the area was 64 children per 1000 affected (6.4%) in 2014—one of the highest rates worldwide [11].

**Figure 1:**
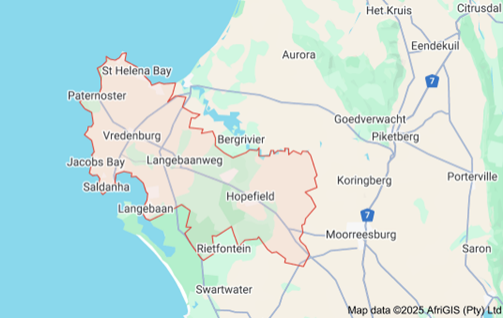
Map of the West Coast region, South Africa.

The study location (Figure 1) presents unique context where Black and mixed-race women in South African communities face a confluence of social and structural challenges that exacerbate the risk of perinatal alcohol use and HIV [37]. A legacy of the apartheid era, the *dop* system—where laborers were paid with alcohol—contributed to a culture of heavy alcohol use, further reinforced by informal local bars, called *Shebeens*. Given the significant contextual and cultural drivers of alcohol use in PLW in this setting, we will prioritize community input and coadaptation of the intervention to optimize intervention fit and relevance.

This work is being carried out in partnership with the Foundation for Alcohol-Related Research (FARR), which has a longstanding community service presence in Saldanha Bay since 2012. We are leveraging this strong community presence to facilitate reaching this hard-to-reach population, build trust, and deliver services in a way that is responsive to their needs and grounded in longstanding local relationships**.**

### Study Aims and Approach

**Figure 2:**
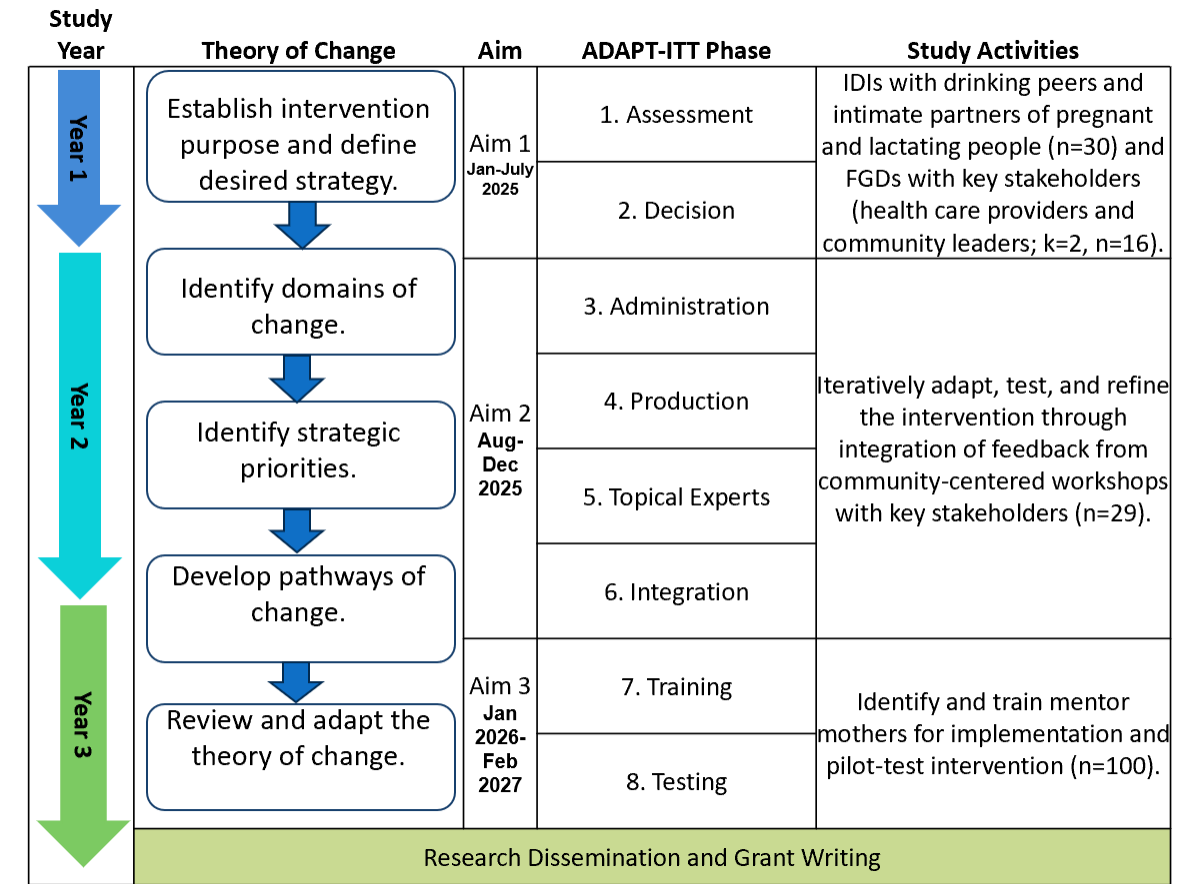
Study research aims and activities mapped on the ADAPT-ITT and theory-of-change frameworks. FGD: focus group discussion; IDI: in-depth interview.

The study aims (Figure 2) are as follows:

- Assess the drivers of perinatal alcohol use from the perspective of key individuals who influence behaviors (drinking peers, intimate partners, community leaders, health care providers) through a mixed methods assessment to identify barriers to and facilitators of alcohol use reduction.
- Adapt the MM model by collaborating with stakeholders and refining the “Mentor Mothers Plus” (MM+) intervention, integrating culturally and contextually appropriate alcohol reduction strategies.
- Evaluate the intervention’s feasibility and acceptability by conducting a pilot randomized controlled trial (RCT) with 100 PLW using alcohol and determine the intervention’s preliminary impact on alcohol use and HIV prevention and treatment behaviors.

### Qualitative Research

We conducted in-depth interviews (IDIs) with partners, as well as peers and family members of pregnant women who drink alcohol. We also conducted focus group discussions (FGDs) with health care and community stakeholders to assess the drivers of perinatal alcohol use from the perspective of key individuals who influence behaviors. We have completed IDIs and FGDs with PLW previously [9,10]. We conducted concurrent mixed methods research with key stakeholders of PLW who use alcohol to explore facilitators of continued alcohol use in pregnancy at the interpersonal, community, and health care facility levels, with the aim of identifying and assessing intervention needs and opportunities (step 1 of ADAPT-ITT).

#### Inclusion Criteria (Formative Stage)

We identified intimate partners and drinking peers (drinking partners, who include friends and family members) of PLW to examine interpersonal and social facilitators of alcohol use while pregnant. In addition, we engaged health care providers working with PLW (nurses, midwives, and HIV counselors) and local leadership (influential community and religious figures) in FGDs to identify facility-, provider-, and community-level barriers to addressing alcohol use in this population (Table 1).

**Table 1:** Description of the purpose, sample, and content of data collection in aim 1 of the MM+^a^ study.

Study methods	Population: intimate partners and peers	Health care population: health care providers and community leaders
Purpose	Understand interpersonal factors contributing to alcohol use among PLW^b^ Understand how alcohol use and HIV risk intersect among PLW Identify support from partners or peers Identify points of intervention	Assess how maternal health care providers address alcohol use in pregnancy as well as access to HIV prevention and care services Identify points of intervention
Sample	Drinking partners (n=15) Intimate partners (n=15)	Health care workers (k=1, n=8) Community and religious leaders (k=1, n=8)
IDIs^c^/FGDs^d^	Firsthand experience of using alcohol with PLW or peers Perceptions of alcohol use in pregnancy Perceived facilitators and barriers to reducing alcohol use in pregnancy Other HIV risk behaviors	Perspectives on alcohol use among PLW Current interventions to address alcohol use among PLW (for providers) Facilitators and barriers to reducing alcohol use in pregnancy and HIV intervention uptake in PLW
Survey measures	Sociodemographic data	—^e^

^a^MM+: Mentor Mothers Plus.

^b^PLW: pregnant and lactating women.

^c^IDI: in-depth interview.

^d^FGD: focus group discussion.

^e^Not applicable.

Trained researchers conducted semistructured IDIs and FGDs using interview guides in Afrikaans and English. All materials were drafted in English and translated into Afrikaans by experienced team members. Data collection for this aim includes (1) semistructured IDIs with drinking and intimate partners of PLW who use alcohol, (2) FGDs with health care providers and community leaders, and (3) concurrently collected brief surveys administered to IDI participants to gather sociodemographic and behavioral data, including perceptions and practices regarding alcohol use during pregnancy (eg, proportion of providers assessing alcohol use at visits, proportion of partners supportive of alcohol use during pregnancy). For qualitative analysis, we will focus on salience-based saturation [38], identifying key themes mentioned by two or more participants.

FARR community-based staff used purposive sampling and snowball sampling for recruitment of drinking partners and intimate partners of PLW who use alcohol. Data collectors received training on sensitive interviewing dealing with distressed participants and referrals.

#### Eligibility Criteria and Key Informant Recruitment

Health care providers were purposively selected from Saldanha Bay municipality clinics and the Vredenburg Provincial Hospital. Community leaders were recruited with help from our Community Advisory Board (CAB), clinic staff, government leaders, social workers, and FARR. An IDI participant must identify as an intimate partner, friend, or family member of a PLW who used alcohol during pregnancy. Participants include adults (≥18 years old) in professional or community roles related to HIV care, maternal health, or alcohol use or in community roles, such as elders, members of faith-based organizations, or civil society representatives, able to provide informed consent. For IDIs with partners, friends, and family members, participants included adults (≥18 years old) living in the study community who reported drinking with a pregnant woman now or in the past. Recruitment was facilitated by the community-based partner through local networks and snowball referrals.

IDIs and FGDs were held in private community spaces or at FARR offices to ensure confidentiality. Participants were reimbursed for their time, inconvenience, and expenses they may have incurred due to being interviewed, with a grocery voucher for a local store. For the FGDs and IDIs, we used rapid qualitative analytic methods. Audio recordings, detailed notes, and transcripts were reviewed by researchers trained in qualitative methods (authors DD and ZP) and fluent in Afrikaans. Data review followed a structured extraction template organized around key domains relevant to intervention adaptation (eg, experiences of people living with HIV [PLWH], provider perspectives, and contextual barriers and facilitators). Data were then transcribed and translated, and two additional analysts (authors ZE and AM) independently populated the template as second reviewers, after which findings were reconciled to resolve discrepancies. The reconciled data were then summarized and cross-checked by the broader study team, including study leadership, to ensure analytic rigor and validity. Extracted data were examined for consistency, key themes, and areas requiring clarification, with opportunities for member checking with community participants to enhance credibility. The resulting thematic findings informed the content presented in the consensus-building workshop and guided adaptation of the intervention.

#### Community Workshop

We applied the community-based participatory action research (CBPAR) principles through three sequential activities to refine and adapt the MM model:

- Adaptation of the MM model through collaborative consensus-building workshop. In August 2025, we conducted a collaborative consensus-building workshop with 29 stakeholders in Saldanha Bay to adapt the MM model (step 3 of ADAPT-ITT). Formative findings from aim 1 and the essential components of the existing MM model (content and literature) were reviewed in a participatory 1-day workshop with health care providers, social workers, civil society, women with lived experience, and community leaders recruited by FARR. The workshop was cofacilitated by the study team with FARR, in both English and Afrikaans, depending on participants’ preferences. Feedback and comments were used to collaboratively adapt key components of the intervention. The study staff obtained consent from participants using English or Afrikaans consent forms. Participants were reimbursed for the time, travel, and expenses. Following the workshop, the adapted MM+ intervention will be iteratively pilot-tested (steps 4-6 of ADAPT-ITT).
- Development of draft MM+ model (August-December, 2025). The research team will use the updated draft intervention and qualitative findings from the workshop to produce a draft ready for pilot-testing among pregnant women and develop study materials, including consent materials, standard operating procedures (SOPs), monitoring and evaluation indicators and tools, and research related instruments (survey and SOPs for blood tests) in preparation for piloting of the MM+ intervention in aim 3.

#### Analytic Approach

We applied rapid qualitative analysis methods for the consensus-building workshop. Notes were reviewed by trained researchers to capture key points from group discussions. Participants focused on core intervention components: who should be included, what content to address, where sessions should take place, and how/when delivery should occur. Each group presented its recommendations to the larger workshop, where ideas were compared and discussed to identify approaches most appropriate for the community context [39,40]. We used findings to inform adaptation of the MM model and development of finalized manuals for the pilot study.

### Study Design for the Pilot RCT (January 2026-February 2027)

We will identify and train 3-5 mentor mothers from the community. Inclusion criteria for mentor mothers include women (≥25 years old) in the community with lived experience who are either on PrEP or have HIV and are on ART and who reported reducing or stopping using alcohol during pregnancy. Mentor mothers will be trained on the adapted MM+ curriculum and MI techniques (step 7 of ADAPT-ITT). We will include 10-15 providers (midwives, nurses, and counselors) to be part of the MM+ training and support the integration of MM+ in three antenatal care (ANC) clinics. Concomitant ante- and postnatal care, including HIV testing and ART and PrEP provision, will be provided to all women regardless of study allocation.

Following training, trained study staff in three local clinics and surrounding communities will recruit and enroll 100 pregnant women who report recent (past 2 months) alcohol use to evaluate the effectiveness and feasibility of the MM+ intervention in reducing alcohol use in PLW, PrEP use in PLW without HIV, and ART adherence in PLW with HIV (step 8 of ADAPT-ITT). Participants will be randomized in a 1:1 ratio to the MM+ study arm or enhanced standard of care (SOC). SOC is focused on HIV counseling by peers, without alcohol messaging. If we are unable to identify 100 women in the three select clinics and communities, we will expand to additional communities and clinics.

### Description of the MM+ Intervention

**Figure 3:**
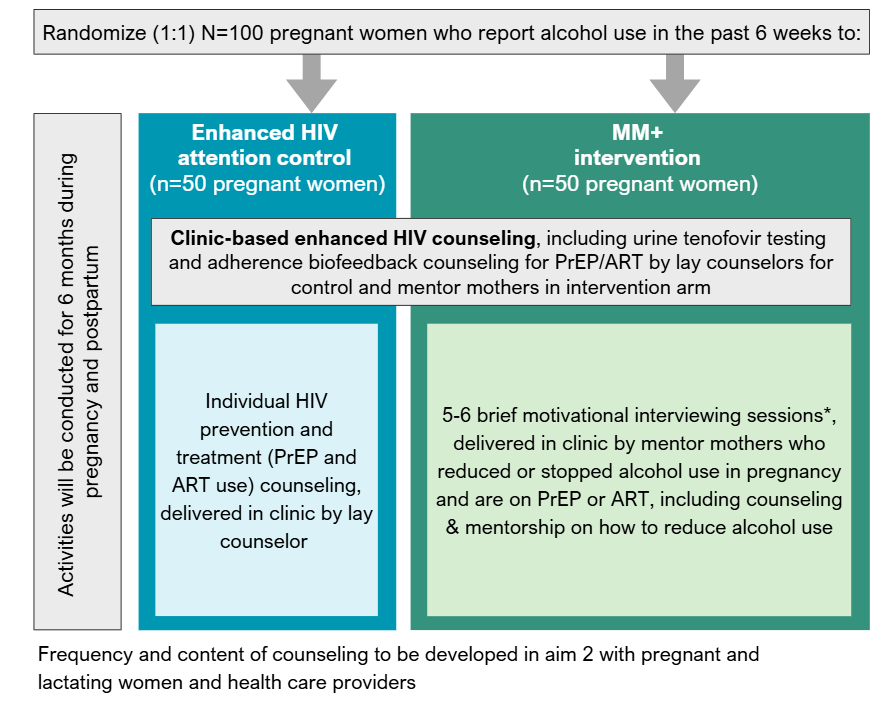
Randomization and allocation of intervention and enhanced standard of care in Mentor Moms+ Study, South Africa.

MM+ will involve mentor mother delivery of brief MI sessions individually over a series of clinic-based visits over a 6-month period during pregnancy and postpartum. Although the exact number of mentor mother sessions, the finalized intervention content, and the full menu of HIV prevention services offered will be finalized during the community workshop, based on the dose from previous MI RCTs in South Africa [41,42], we anticipate no fewer than five to six 15-20–minute MI sessions focused on the benefits of reducing alcohol use during pregnancy and managing HIV risk among women without HIV and the importance of ART use and viral suppression for reducing the risk of vertical HIV transmission. Participants in both study arms will receive enhanced HIV prevention counseling integrated into routine ANC visits, including PrEP and ART biofeedback adherence using urine tenofovir (TFV) tests that are point-of-care inexpensive tests that provide feedback on the recent use of PrEP or ART in the past 72-hours [43-45]. In the intervention arm, these services will be provided by a trained study nurse. See Figure 3 for a breakdown of the anticipated intervention structure.

#### RCT Outcomes

The RCT primary outcome is the preliminary effectiveness (comparing the MM+ arm to the enhanced HIV attention control arm using intention-to-treat analysis) defined as a reduction in alcohol use via biomarker-confirmed self-reported and biological samples (comparing baseline to 6 months in phosphatidylethanol [PEth] blood samples).

Secondary outcomes include (1) PrEP initiation and adherence (via patient records and urine TFV testing at 6 months) and (2) ART adherence (via urine TFV testing and self-report). In addition, we will evaluate three of the eight implementation outcomes, as defined by Proctor et al [46]: feasibility, acceptability (including participant satisfaction with the intervention), and fidelity. We will also track any social or health harms in the study and relatedness to the study intervention through study surveys and review of medical records. Table 2 describes how these outcomes will be measured. We will track session attendance, as well as ART and PrEP adherence, at each study visit.

**Table 2:** Description of aim 3 implementation outcomes.

Evaluation parameter	Description	Methods	Frequency
Theoretical model	Evaluate changes in alcohol use and HIV prevention or treatment (eg, PrEP^a^ and ART^b^ use) using the theory-of-change model measuring change and which sociodemographic and behavioral characteristics are associated with change in order to understand any subpopulations receptive toward the strategic priorities and pathways of change identified by our model.	Quantitative surveys at each visit and biomarker data	Baseline and 6-month follow-up
Participants’ self-reported fidelity	Evaluate completeness of the intervention through a checklist assessing whether specific topics were covered during intervention sessions, including an assessment of the quality of intervention interactions, where participants will rate implementer engagement during intervention sessions.	Quantitative surveys at each visit and biomarker data	Baseline and 6-month follow-up
Acceptability and uptake of and satisfaction with the intervention	Evaluate the organizational, provider, and PLW^c^ acceptability of the intervention, including the number and percentage of sessions attended and IDI^d^ feedback following the sessions. Success would be >80% of sessions attended with very good/good feedback postsessions.	Mixed methods	Postimplementation mixed methods: 6-month survey (all participants), followed by IDIs with 20 providers and 20 participants
Fidelity to training assessment	Evaluate sessions (20% recorded and reviewed by the principal investigator) and fidelity to training using a checklist of key parameters. Success would be >80% of sessions supervised following training SOPs^e^.	Monitoring forms: comparison of SOPs and audio-recorded MM+^f^ sessions	Postimplementation mixed methods: 6-month survey (all participants), followed by IDIs in 20 providers and 20 participants
Feasibility of the intervention	Evaluate organizational, provider, and PLW views on the feasibility and scalability of the implementation of the intervention.	Mixed methods	Postimplementation mixed methods: 6-month survey (all participants), followed by IDIs in 20 providers and 20 participants

^a^PrEP: pre-exposure prophylaxis.

^b^ART: antiretroviral therapy.

^c^PLW: pregnant and lactating women.

^d^IDI: in-depth interview.

^e^SOP: standard operating procedure.

^f^MM+: Mentor Mothers Plus.

#### Outcome Data Ascertainment

Participants will be followed for 6 months. During this time, they will have three study visits, in which they will self-report alcohol use and PrEP or ART initiation or continuation to study staff. At each study visit, we will ascertain dried blood spots for assessment of recent alcohol use, via PEth testing (PEth<20 ng/mL represents light or no consumption [47]) and AUDIT-C, a validated survey of alcohol use (0=abstinence, >0=recent alcohol use). Participant medical files will be reviewed at the end of the study to ascertain birth outcomes (including any pregnancy loss or stillbirth), reported harms, and PrEP and ART prescription. At each study visit, participants will provide a urine sample to the study staff for analysis of recent PrEP or ART use using urine TFV testing (of recent adherence in the past 72 hours). Urine TFV results will show recent adherence and will be evaluated in conjunction with viral loads (VLs). For those on ART, we will review their files for VLs to evaluate VL suppression at baseline and end of the trial (VL<200 copies/mL). If VLs are unavailable, we will track them online with the national health laboratory service.

### Enhanced Standard of Care

Given that existing MM models focus on vertical HIV transmission and HIV prevention and treatment, and our primary aim is to understand how inclusion of alcohol reduction strategies reduces alcohol use and improves adoption of ART/PrEP use, we will use enhanced SOC with the same time frame for the intervention (eg, two to three sessions during antenatal and two to three sessions during postpartum periods). This will consist of educational counseling delivered by peers (mentor mothers) on HIV risk, partner testing modalities, and PrEP/ART urine assay testing, followed by real-time in-clinic adherence counseling delivered privately (one on one) by mentor mothers (separate from intervention mentor mothers to avoid contamination), all coinciding with routine ANC visits when possible.

Benefits of the study include counseling and support from lay counselors (in SOC) and mentor mothers (in the intervention) to address alcohol use and HIV prevention and care. Risks include unintended disclosure of alcohol use or HIV status by providers or staff and potential breaches of confidentiality that could cause social harms, including IPV. We will train staff and providers to ensure they are aware of these risks and do not share details of participants’ status outside of the study. We will monitor all social and other study harms throughout the study through regular surveys and review of medical records.

### RCT Inclusion Criteria

Women who are ≥16 years old, with a confirmed pregnancy; who report alcohol use during pregnancy (in the past 2 months); who live within 20 km of the study facility; and who are able and willing to consent to study participation are eligible to participate.

One trained research assistant based at the ANC clinic will screen and obtain informed consent from PLW during their routine ANC visits using a set script for screening participants. We have received a waiver of parental consent for adolescents aged 16 and 17 years.

We will randomize the enrolled participants who have provided informed consent to the MM+ intervention or to enhanced HIV attention control in a 1:1 ratio. Randomization will be performed immediately following enrollment using dynamic permuted blocks with REDCap randomization sequentially. Trained researchers will not have access to the random allocation sequence. The intervention is nonblinded.

All study procedures will be in a private space in the Saldanha Bay municipality clinics, including screening, review, and signing of consent forms, surveys, and other procedures. This space will be dedicated research space given to the Human Science Research Council (HSRC) and FARR teams. We will ask pregnant women who may be interested in the study to see the study staff (in the study room) at the end of their visits or to get an appointment for another day to return to the clinic for study enrollment (if eligible).

### Analysis Plan

#### Outcomes

The implementation outcomes include feasibility (provider, organizational, and participant feedback), acceptability (number of sessions attended, feedback on content), and fidelity to study SOPs on MM+. The safety outcomes include IPV and pregnancy and birth outcomes (including preterm birth, birth weight, and miscarriage or stillbirth and infant survival) [48,49].

#### Power Calculations

We calculated power based on a 20% or more reduction in alcohol use in the intervention arm compared to no reduction (or minimal <5% reduction) in the control group. By enrolling 100 pregnant women (n=50, 50%, per arm), our study will be adequately powered at >80% for all primary effectiveness outcomes of feasibility and reduction in alcohol use. In qualitative work, the sample size is determined by the number of interviews expected with a specific population to reach thematic saturation [50,51].

#### Qualitative Data Analysis

We will transcribe, translate, and import postintervention qualitative data into a software package, and a team of three researchers with expertise in qualitative research methods will code the data using a hybrid inductive-deductive approach. We will measure interrater reliability to ensure consistent code application between interviewers; this process will be iterative, and code definitions will be refined until application is consistent across coders. We will develop code summaries and memos for emerging themes using an experienced trained team of qualitative researchers from the HSRC and the San Diego State University (SDSU), who will use open coding to analyze segments of the data, create focused codes, and highlight quotable text. We will use a deductive approach from our theoretical framework on individual-, facility-, and HIV-level facilitators of alcohol reduction or abstinence and PrEP or ART continuation and adherence, allowing inductive codes to emerge. We will use a thematic analysis allowing key issues to emerge from the data. Multiple data sources (participants, providers) will be triangulated to provide a holistic understanding of the intervention acceptability [52]. We will use the COREQ (Consolidated Criteria for Reporting Qualitative Research) checklist to ensure positionality, reflexivity, etc, are covered to ensure rigor [53].

#### Descriptive Analysis and Effectiveness Outcomes

We will use basic measures (proportions, means and SDs, medians and IQRs, as appropriate) and graphical displays to describe distributions of key variables. Bivariable analyses will use Student's *t* tests (replaced by Mann-Whitney U tests for nonnormal distributions) or chi-square tests (replaced by exact tests for sparse data), as appropriate. We will compare study arms’ descriptive statistics without tests of statistical significance. Comparisons of randomized groups’ primary analyses will be by intention to treat, with secondary analyses based on intervention uptake. Specifically, we will evaluate reductions in alcohol use from baseline to 6-month follow-up in PEth levels, with confirmation of self-reported AUDIT-C scores compared by study arm. For secondary HIV outcomes, we will evaluate ART and PrEP adherence by study arm using urine TFV levels for recent adherence and VLs at 6 months for women on ART.

All statistical tests will be two-sided at α=.05; all effect estimates will be reported with 95% CIs. For both aims, we propose a priori subgroup analyses by age and HIV status. For subgroup analyses, effect modification will be assessed from the fully adjusted model on the additive scale by calculating the relative excess risk due to interaction [54] and on the multiplicative scale by examining the interaction term in regression models. Data will be analyzed in Stata version 15.

We will track missing variables in our monthly quality control and follow-up on all missing exposures and outcomes at each visit. We will conduct sensitivity analyses, including multiple imputation, to include evaluation outcomes and exposures from missing data and incomplete retention. Our retention efforts will focus on retention in both groups to ensure that there is no differential loss to follow-up. Analysis of implementation outcomes will entail descriptive summarization of acceptability, feasibility, and fidelity of MM+, including for women of different socioeconomic statuses or education levels.

Data management procedures will be developed with the statistical analysis plan and available upon demand to study investigators.

#### Mixed Methods Analyses

We will conduct an explanatory sequential mixed methods design [55], collecting and analyzing quantitative data first (surveys, monitoring forms), followed by qualitative interviews with a purposively selected subsample of health care providers and participants to expand on findings from the quantitative phase.

### Ethical Considerations

We have obtained approval from relevant institutional review boards (University of California, Los Angeles [UCLA] IRB#24-0130, HSRC REC #2/31/01/24). Prior notice will be given, and informed consent will be sought and obtained from all study participants. All participants will go through an informed consent process. The study staff will provide information for those who are interested in being tested but not interested in study participation. All study participation is strictly voluntary, and participants can refuse specific procedures or further study participation at any time.

Study staff members who will have contact with participants will receive training on the protection of human subject research before engaging in study-related activities. Additionally, the staff will undergo Good Clinical Practices (GCP) training, as required. Evidence of ethics training will be submitted with every ethics approval application and maintained throughout the study.

All potential participants will read the informed consent form (ICF) or have the ICF read to them. The ICF will be available in relevant local languages (eg, Afrikaans, isiXhosa) and English, and participants will complete the informed consent process in their preferred language. Each participant will be given an opportunity to ask questions about study participation. If a participant agrees to participate in the study, the study staff will obtain written consent. After consenting to participate, the participant may voluntarily withdraw from the study at any time and can choose not to have their responses submitted to the study team. Study participation is voluntary, and we will ensure ongoing respect of participants throughout the research process through ensuring confidentiality and privacy principles.

To ensure confidentiality, all data will be coded by participant number. Data recorded on paper will be kept in locked cabinets with access only to members of the research team. There will be strict limited access to electronically stored data, using password protection. Research records will be kept confidential following national regulations. Every participant’s name or other personal identifiers will not be included with specimens sent to the laboratory, which will be identified only by a code number. Interviewers and support staff will be trained on procedures for maintaining confidentiality.

We will use phone calls and text messages for follow-up to improve retention. Text messages will be neutral and not contain personal information about the participants (eg, appointment reminders will say, “Your appointment is on Tuesday at 3:00 p.m.”). At each visit, staff will ask about social harms, complete a detailed report, link the participants to community resources for support, and report social harms to the ethics review board. If multiple social harms are observed, the study procedures and text message content will be reviewed by the study team and CABs and recommended changes submitted to regulatory bodies for review.

All data collection procedures, regardless of the source, will follow standardized procedures outlined in the *Manual of Operating Procedures*, with individual activities guided by SOPs. RCT data (aim 3) will be collected by trained study interviewers using REDCap electronic software, which is Health Insurance Portability and Accountability Act (HIPAA)– and Protection of Personal Information Act (POPIA)–compliant for research studies. The interviewers will ask questions and complete the responses using a handheld tablet with internet connectivity for rapid data access and quality control.

The Data Safety and Monitoring Board (DSMB), which will comprise experts in the field of HIV and substance use in pregnancy, will be formed in the first year of the study and will meet quarterly to evaluate safety outcomes and data monitoring. A CAB was formed in 2025 that includes pregnant individuals, health care providers, and community leaders from Saldanha Bay and has held two meetings to date. CAB members are supporting the study by providing input, guidance, and feedback on the study’s methodology and implementation throughout the study period, ensuring community perspectives are considered.

## Results

We launched our formative work in January 2025 with community research in Saldanha Bay. We conducted 2 FGDs with health workers (n=6) and community leaders (n=7), as well as 31 IDIs with partners and peers of PLW who reported alcohol use during pregnancy. We have conducted the initial rapid analysis of the results. Participants reported that PLW have strong motivations to reduce alcohol use for their child’s health but face multiple barriers, including misconceptions about the safety of alcohol in pregnancy, poverty, trauma, IPV, social pressure to drink to connect with peers, and stigma. Alcohol use was frequently linked to financial stress (particularly around the need to support a child), an unplanned pregnancy, and poor mental health. Preferred support strategies emphasized nonjudgmental, peer-based mentorship, family and partner engagement, and involvement of faith and community networks and leaders. Additional gaps included stigma surrounding HIV prevention (PrEP awareness, access, and use were limited) and contraceptive services. Communities recommended that mentor mothers be women with lived experience who are empathetic, discreet, and culturally relatable, with structured training, supervision, and referral linkages.

Following rapid analysis, a community workshop was convened in August 2025 with 29 stakeholders, including community leaders, health professionals, social workers, and people with lived experience, to refine the MM+ intervention. Participants reinforced the importance of mentor mothers as trusted, trained women with lived experience, and highlighted priorities, such as clear, culturally relevant education on alcohol use, HIV prevention, and family planning; trauma and IPV support; and flexible delivery across home, community, and group settings.

The next steps include identification and training of mentor mothers, finalization of intervention materials, and preparation for implementation of the RCT.

## Discussion

### Summary

Our study addresses the overlapping challenges of alcohol use and HIV among PLW in a high-burden setting by developing and piloting MM+, an adapted, community-informed version of the MM model. Through a phased, participatory approach grounded in the ADAPT-ITT framework and community-based research principles, MM+ integrates alcohol reduction with HIV prevention and treatment using a HIV seroneutral approach. The intervention is designed to reflect the real-world needs of PLW, their partners, health care providers, and communities.

Although PLW face increased risks related to both HIV and alcohol use, few community-informed interventions have directly addressed these intersecting vulnerabilities. Existing brief interventions to reduce alcohol use during pregnancy have shown limited and short-lived impact, partly due to minimal follow-up and lack of biomarker validation. Evidence from South Africa demonstrates that integrating peer mentor mothers into care structures can improve maternal health outcomes and reduce vertical HIV transmission through task shifting, whereby trained peer mothers deliver counseling and support within routine and community-based care. These MM models, pioneered by mothers to mothers, have primarily focused on preventing mother-to-child HIV transmission and improving maternal-child health, with only limited adaptation to address alcohol or substance use. Although alcohol has not been a central component of MM interventions, prior adaptations illustrate the flexibility and effectiveness of this platform for addressing broader maternal-child health priorities.

Building on this foundation, our study applies the ADAPT-ITT framework to systematically tailor the MM model to explicitly incorporate alcohol use reduction alongside HIV prevention and treatment. By leveraging an established, trusted, and evidence-based delivery platform, we aim to ensure that the adapted intervention is both responsive to community needs and feasible for integration into existing health systems. Through a structured participatory process using rapid qualitative methods, we demonstrated the feasibility of adapting the MM model for the South African context. Findings from formative interviews and a consensus-building workshop highlighted strong stakeholder support for tailoring content, delivery modalities, and session structures to local realities. Participants emphasized the value of integrating women with lived experience as peer mentors, delivering sessions in accessible community-based venues, and addressing both health-related and psychosocial challenges faced by PLW.

### Strengths

This study has several notable strengths. First, it builds on an established, evidence-based MM platform that has been successfully implemented to reduce vertical HIV transmission, increasing the feasibility and sustainability of our adaptation. Second, the intervention is community informed and peer delivered, which enhances cultural relevance, trust, and the likelihood of uptake among PPW. Third, the use of the ADAPT-ITT framework ensures a systematic and theory-driven approach to intervention modification, increasing the rigor of the adaptation process. In addition, incorporating biological markers of alcohol use alongside self-reported measures strengthens the validity of outcome assessment and addresses limitations of prior studies. Finally, the focus on integrating HIV prevention and alcohol reduction within maternal and child health platforms reflects a holistic approach that aligns with existing health system priorities.

### Limitations

Several limitations should be acknowledged. As an adaptation and implementation study, findings will be context specific, and generalizability beyond the study setting may be limited without additional testing. The reliance on peer-delivered counseling may pose scalability challenges in regions with limited MM program coverage or constrained human resources in the current South African context. Although biomarkers improve accuracy, they may not fully capture patterns of alcohol consumption, and social desirability bias in self-reports could remain. Finally, the intervention’s impact on longer-term outcomes, such as sustained alcohol reduction and HIV incidence, will require extended follow-up beyond the study period.

### Conclusion

This pilot trial will assess the feasibility, acceptability, and preliminary impact of MM+, while also generating insights into personal and structural barriers to care. We hypothesize that the intervention will be acceptable among pregnant women and providers and will reduce alcohol use during pregnancy, while also improving access to HIV prevention and care. Findings will inform future scale-up efforts, with the goal of improving maternal and child health through a practical, scalable strategy in high-burden and low- and middle-income settings.

## Data Availability

Data will be available for review upon request to study investigators. The trial protocol and statistical analysis plan can be accessed via the study principal investigators. All data will be disseminated during the study period to the community, participants, and stakeholders through regular meetings. Primary and secondary outcomes, including formative research results, will be communicated through conferences and peer-reviewed manuscripts during and following the trial period.

## Appendix

Multimedia Appendix 1 Focus group topic guide.

Multimedia Appendix 2 Interview guide.

Multimedia Appendix 3 Peer-review report by PPAH-Population and Public Health Approaches to HIV/AIDS Study Section (National Institutes of Health, USA).

## Abbreviations

ANC: antenatal care
ART: antiretroviral therapy
CAB: Community Advisory Board
FARR: Foundation for Alcohol-Related Research
FASD: fetal alcohol spectrum disorder
FGD: focus group discussion
HSRC: Human Science Research Council
ICF: informed consent form
IDI: in-depth interview
IPV: intimate partner violence
MI: notivational interviewing
MM: Mentor Mother
MM+: Mentor Mother Plus
PEth: phosphatidylethanol
PLW: pregnant and lactating women
PrEP: pre-exposure prophylaxis
RCT: randomized controlled trial
SOC: standard of care
SOP: standard operating procedure
TFV: tenofovir
VL: viral load
