# Supplementary material for: Community-Based Adaptation and Evaluation of a Peer-Led Intervention to Address Alcohol Use and HIV in Pregnant and Breastfeeding Women in South Africa: Protocol for the “Mentor Mothers Plus” Randomized Control Trial
Source: JMIR Res Protoc. 2025 Dec 18;14:e78856. doi: 10.2196/78856 (PMC12757709; doi:10.2196/78856)
Supplement: Multimedia Appendix 1 [file resprot_v14i1e78856_app1.pdf]

**Mentor Moms+: Focus Group Discussion Guide for Healthcare Providers:**

**Providers:** Midwives, Nurses, Social Worker/Psychologist, Counsellors, Managers (OM or Sister in Charge)

## Understanding Alcohol Use in Pregnancy

### Welcome and Introduction

- Start with a warm welcome and a brief introduction about yourself, the purpose of the focus group, and the organizations involved.
- Highlight the importance of understanding alcohol use among pregnant and breastfeeding women from a healthcare perspective.

### Consent and Confidentiality Reminder

- Remind participants about the consent form they have signed and reassure them about confidentiality and the voluntary nature of their participation. Invite any last-minute questions before beginning.
- No one can share information about who is in this meeting or what is said outside of this room.

### Discussion Segments

### Segment 1: Demographics and Icebreakers

- Could each of you share how long you've been providing (or working in) antenatal care?
- What is your favourite part of working with pregnant women?
- What are some of the challenges of working with pregnant women?

### Segment 2: Research prioritization exercise

We will use the following method to identify barriers and facilitators for harm reduction and improved HIV care in pregnant women in your community.

Each person gets an opportunity barriers and facilitators (on a sticky note or tablet) and then share ideas, clarify their ranking of the ideas, then vote on their priorities (from most important to least important).

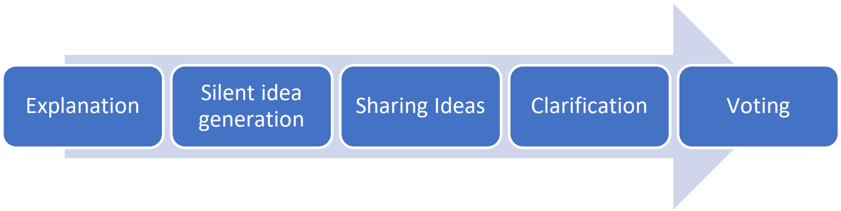


### Segment 2: Patterns of Alcohol Use Among Pregnant Women

- Thinking of the population of women you serve, what have you noticed about alcohol use among pregnant women?
- Tell me more about what your community perceptions (ideas) about alcohol consumption during pregnancy? (Probe: Can you share any specific examples?)
    - Have you noticed women in your community altering their drinking habits during pregnancy? What changes have you observed?

**Segment 3: Healthcare Provider Observations and Conversations**

- What are healthcare provider views about pregnant women who use alcohol?
- Could you share how alcohol use in pregnancy is addressed during routine care in your clinic?
    - How do you record alcohol and drug use in pregnancy? (Probe: every visit or just first ANC? Follow up of alc+ women)
- What are the common reasons women give for continuing to drink during pregnancy? (Probe: breastfeeding myths, health/beauty of baby, stress)
- How do you approach these conversations with pregnant women? (Probe: do you discuss alcohol use with others (family members, partners, etc)
- How do you support women with problem drinking/drug use in your community? (Probe: referral to counselling, where, how, accessibility, etc)

**Segment 4: Socio-cultural Influences on Perinatal Alcohol Use**

- What messages about alcohol use during pregnancy are prevalent in your community? How do these messages compare to what is conveyed in clinics?
- In your experience, how do partners influence a pregnant woman's alcohol consumption?
- In your experience, how do peers (friends, family) influence a pregnant woman's alcohol consumption?
- What support systems exist for pregnant women who use alcohol? What support systems are needed to help women reduce alcohol use during pregnancy?

**Segment 5: HIV Prevention & Treatment**

- How does alcohol use among pregnant women impact their decision-making regarding HIV prevention or treatment?
- From your perspective, what are the most effective HIV prevention methods for pregnant women in your community?
- From  your perspective, how to women living with HIV access and stay on ART? How might alcohol impact on ART use in WLHIV?

**Segment 6: Intervention recommendations**

- From your perspective, what support might help women ensure they have healthy pregnancies and infants?
- What could **you do** better (or you clinic) to improve care for pregnant women?
- Specific to alcohol or drug you, what support might help women ensure they have healthy pregnancies and infants?
- What could you do better (or you clinic) to improve care for pregnant women who drink alcohol or use drugs?
- Mother2Mothers is a programme that provides HIV counselling and support for pregnant women living with HIV over time by peers of women living with HIV.

    - How do you think we could adapt this mentor mothers model for your clinic population?
    - From your perspective, how could we use mentor mothers for alcohol and drug use?
    - Do you think women would be willing to participate in this intervention (Why/why not)?
- How could you work with a mentor mothers program to ensure success?

**Closing**

- **Wrap up the discussion by thanking participants for their valuable insights and reiterating the importance of their contributions to developing effective health services.**

**Tips for Facilitating the Discussion**

- Encourage open dialogue among participants, allowing them to respond to each other's comments.
- Use probing questions to delve deeper into specific points raised by participants.
- Manage the discussion to ensure all participants have the opportunity to share their perspectives.
- Take detailed notes or record the session (with consent) for accurate documentation of the discussion.
