# Supplementary material for: Community-Based Adaptation and Evaluation of a Peer-Led Intervention to Address Alcohol Use and HIV in Pregnant and Breastfeeding Women in South Africa: Protocol for the “Mentor Mothers Plus” Randomized Control Trial
Source: JMIR Res Protoc. 2025 Dec 18;14:e78856. doi: 10.2196/78856 (PMC12757709; doi:10.2196/78856)
Supplement: Multimedia Appendix 2 [file resprot_v14i1e78856_app2.docx]

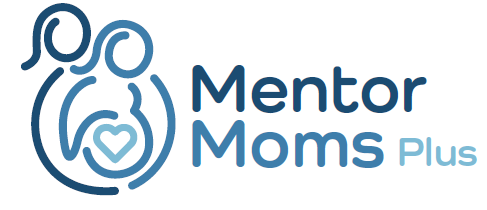


**Mentor Moms+: Semi-structured Interview Guide for Drinking Peers of Pregnant Women**

**Introductions:** Hello, my name is ______________________. I am a research assistant and on behalf of HSRC and I would like to ask you some questions to better understand decision around alcohol use in pregnancy in your community. We want to understand patterns of drinking among pregnant and breast-feeding women and identify individual, interpersonal, and social factors that may influence these issues.

You have been referred to us because you have a pregnant friend. We are interested in learning more about your own experiences with your friends or family who may use alcohol or drugs during pregnancy. Your responses will help us to develop and deliver health services tailored specifically to the needs of this population. In reference to the consent form which you have read/has been read to you, and already signed Do you have any questions for me before we begin?

**Demographics**

1. How old are you?

2. How many children do you have? ____

1. Tell me more about your relationship with the pregnant mum who referred you to this study?
   1. Probes: how long have you known her? How close are you? Living status?

**Patterns of alcohol use among pregnant women**

1. Tell me a bit more about the use of alcohol among pregnant women in your community, circle of friends or family?
2. What is the attitude towards pregnant women who use alcohol in your community? *(Probe: For example, how is a pregnant woman who is drinking in the bar treated?)*

Now I would like to hear about examples from your own friend circle.

1. Tell me about when you and your friends typically drink. Where does this happen? When? With Whom? What and how much? *(probe: do people drink to get drunk?)*
   1. Think about times when friends in your peer group have been pregnant, did they drink during the period between when they became pregnant and when they both found out they were pregnant? (probe: this is referring to those first week/months when one doesn’t realize they are pregnant yet).
2. How did your friend’s drinking patterns change when they learned they were pregnant? *Probe: Did she continue to drink? With you? In the same locations (e.g., bars, taverns, home with you)? The same amount? Did she hide her drinking from anyone? If so, why?*
   1. Was her partner or family aware of their alcohol use during pregnancy? If so, how was this received? *(probe: tell me more about who she drinks with and when? How?)*
   2. What factors influenced your friend’s decision making around continuing to use alcohol or not during pregnancy?? *Probe: how did you feel about this decision? Were you actively engaged in any conversations around this topic? Were there any disagreements around it?*
3. If someone wants to stop drinking alcohol during pregnancy is this something that would be supported by peers in your friend group or family? *Probe: why or why not? How so?*
4. What do you think might be a safe amount, or type of alcohol, to drink while pregnant? *(Probe: if, yes, what is that level and is this a commonly held belief? What types of alcohol may be less harmful?)*
   - 1. What about while breastfeeding?

**Interpersonal and socio-cultural drivers perinatal alcohol use and IPV**

1. What are they things you have heard leaders, family, providers, say about alcohol use during pregnancy? *(Probe: What about from: community leaders? Health clinics? Peers? Parents/elders? How about warnings on bottles of alcohol?)*
2. What are the factors that make it difficult for women to stop or reduce using alcohol during pregnancy in your community? *(Probe: “why does [whatever they mention] make it easy for women to continue to use alcohol?”*
3. What support might help pregnant women to reduce or stop drinking while pregnant and breastfeeding? Who could provide support?
4. What do you think about having mothers in your community who have children (and may have reduced/stopped drinking or using drugs) provide support to pregnant women who drink alcohol? (*Probe: What kinds of sessions? Support? Counseling (individual or group), at home or in community)*
