# Supplementary material for: Community-Based Adaptation and Evaluation of a Peer-Led Intervention to Address Alcohol Use and HIV in Pregnant and Breastfeeding Women in South Africa: Protocol for the “Mentor Mothers Plus” Randomized Control Trial
Source: JMIR Res Protoc. 2025 Dec 18;14:e78856. doi: 10.2196/78856 (PMC12757709; doi:10.2196/78856)
Supplement: Multimedia Appendix 3 [file resprot_v14i1e78856_app3.pdf]

SUMMARY STATEMENT

(Privileged Communication)

PROGRAM CONTACT:
Dr. Robert Freeman
301443-8820
rfreeman@mail.nih.gov

Release Date: **12/05/2023**
Revised Date:

Application Number: **1R34AA030942-01A1**
Formerly: **1R34AA030942-01**

Principal Investigators (Listed Alphabetically):

ESSACK, ZAYNAB
JOSEPH DAVEY, DVORA (Contact)

Applicant Organization: UNIVERSITY OF CALIFORNIA LOS ANGELES

Review Group: **PPAH**
Population and Public Health Approaches to HIV/AIDS Study Section
AIDS

Meeting Date: **11/15/2023**
Council: **JAN 2024**
Requested Start: **04/01/2024**
Opportunity Number: **PA-21-110**
PCC: **APAF**

Project Title: **A community-based adaptation of a peer-led intervention to address alcohol use and HIV risk in pregnant women in South Africa (Mentor Moms+)**
SRG Action: **Impact Score:38**
Next Steps: **Visit https://grants.nih.gov/grants/next_steps.htm**
Human Subjects: 30-Human subjects involved - Certified, no SRG concerns
Animal Subjects: 10-No live vertebrate animals involved for competing appl.
Gender: 1A-Both genders, scientifically acceptable
Minority: 5A-Only foreign subjects, scientifically acceptable
Age: 3U-No children included, scientifically unacceptable

Project Year	Direct Costs Requested	Estimated Total Cost
1	150,000	200,261
2	150,000	200,261
3	150,000	200,261
TOTAL	450,000	600,784

ADMINISTRATIVE BUDGET NOTE: The budget shown is the requested budget and has not been adjusted to reflect any recommendations made by reviewers. If an award is planned, the costs will be calculated by Institute grants management staff based on the recommendations outlined below in the COMMITTEE BUDGET RECOMMENDATIONS section.

1R34AA030942-01A1 JOSEPH DAVEY, DVORA

INCLUSION ACROSS THE LIFESPAN UNACCEPTABLE

RESUME AND SUMMARY OF DISCUSSION: In this resubmitted R34 application, the investigative team proposes to identify facilitators of alcohol use among pregnant and lactating people (PLP) in South Africa; adapt the mentor mother (MM) model to create MM+ for PLP who use alcohol; and evaluate the feasibility, acceptability and effects on alcohol use, PrEP use and ART adherence of this adapted intervention. The significance of the proposed work is high, based on rigorous prior research on both the base intervention as well as the importance of addressing alcohol use among PLP. Targeting interventions to antenatal care (ANC) clinics also has the potential for broad applicability. The investigative team is very strong, with the needed expertise in epidemiology as well as social behavioral sciences. The proposed work is highly innovative in addressing alcohol use among PLP, although the methods are not overly innovative. The approach has several strengths, including application of the ADAPT-ITT model; use of biomarkers; strong preliminary work; documentation of implementation costs; and sound explanatory sequential mixed methods for Aim 3. Weaknesses, which partially offset the strengths, included missing details on whether mentor mothers would be compensated and whether providers are existing ANC staff; potentially infeasible recruitment targets; insufficient exploration of differences between HIV+/HIV- PLP; and the inability to look at clinic-level factors. The environment is excellent, with the needed resources to carry out the work. Although human subjects' protections and inclusions are largely appropriate, it is unclear why PLP under the age 18 are being excluded. The application is very responsive to the prior critique. At the end of discussion, reviewers expressed that the strengths of the application slightly outweighed its weaknesses, and predicted its likely impact on the field of HIV prevention among PLP was moderately high.

DESCRIPTION (provided by applicant): The syndemic of alcohol use and HIV risk in pregnant and lactating people (PLP) threatens the health of mother, fetus, children and families in South Africa. PLP living with HIV who use alcohol may access antiretroviral therapy (ART) late or disengage with ART care, increasing the risk of vertical HIV transmission. PLP not living with HIV who use alcohol are at increased risk of HIV and may require targeted interventions to receive pre-exposure prophylaxis (PrEP) delivery and adherence counselling. Alcohol use also increases risk of HIV acquisition and poor ART adherence. The "mentor mother" (MM) intervention model is an evidence- based intervention (EBI) with demonstrated success in improving HIV and antenatal care outcomes. The model utilizes a task-shifting approach; positive deviant peers (mothers) deliver interventions to PBFW within and outside of the antenatal clinic. In the present study, we will collaborate with community partners to identify facilitators of alcohol use among PLP in SA. We will then adapt the MM model to create MM+ for PLP who use alcohol to evaluate the feasibility of an alcohol reduction intervention that includes HIV prevention and treatment (serostatus neutral) in a high alcohol using and HIV burdened community. Specific aims include: · Aim 1: Mixed methods assessment of perceptions of alcohol use in pregnancy and facilitators of alcohol reduction in pregnancy among multilevel stakeholders of PLP who use alcohol to identify clinic and community level facilitators that could be used to reduce the use of alcohol during pregnancy through in-depth interviews (IDIs) and brief surveys with drinking peers, intimate partners and focus group discussions with healthcare providers and community leaders. · Aim 2: Adapt and refine enhanced MM intervention (MM+) with potential intervention beneficiaries, providers and community leaders. Use community based participatory approach research principles to iteratively adapt, test and refine the MM+ intervention to integrate alcohol reduction content in one collaborative workshop session. · Aim 3: Evaluate the feasibility and acceptability of MM+ on reduction of alcohol use (primary outcome) and PrEP use (in PLP without HIV) and ART adherence (in PLP living with HIV) (secondary outcomes) in a pilot randomized control trial (RCT) in n=100 pregnant women who currently use alcohol. Primary outcome: Reduced alcohol use following the intervention (at 6m via

phosphatidylethanol [PEth] levels). Secondary outcomes: PrEP and ART continued use at 6m via urine tenofovir levels at 6m. Secondary implementation outcomes: feasibility (provider, organizational and participant feedback), acceptability and fidelity to the intervention SOPs. Our study seeks to effectively reduce alcohol use and improve HIV care outcomes through community engagement and a novel participant informed intervention adaptation and delivery process.

PUBLIC HEALTH RELEVANCE: South Africa has the highest combined prevalence of alcohol use in pregnancy and HIV in the world; pregnant women without HIV require access to effective HIV prevention including oral pre-exposure prophylaxis (PrEP), and pregnant women living with HIV who use alcohol may access antiretroviral therapy (ART) late or disengage with ART prior to or during pregnancy, increasing the risk of vertical HIV transmission. There are no evidence-based, community-informed interventions available to minimize the range of negative health outcomes due to alcohol and HIV in pregnancy. Our innovative study seeks to: · Aim 1: Conduct a mixed methods assessment of alcohol use in pregnancy and facilitators of alcohol reduction in pregnancy among multilevel stakeholders of pregnant women who use alcohol to identify clinic and community level facilitators that could be used to reduce the use of alcohol during pregnancy; · Aim 2: Adapt and refine an evidence-based HIV prevention intervention, Mentor Mothers (MM+), to include perinatal alcohol use and HIV care with potential beneficiaries (pregnant women who use alcohol), healthcare providers and community leaders; · Aim 3: Evaluate the feasibility and acceptability of MM+ on reduction of alcohol use (primary outcome) and PrEP use (in pregnant women without HIV) and ART adherence (in pregnant women living with HIV) (secondary outcomes), as well as feasiblity (among providers), acceptabilty (among pregnant women) and fidelity to the intervention, to inform future research and scaled interventions in South Africa and throughout the region.

CRITIQUE 1

Significance: 2
Investigator(s): 2
Innovation: 3
Approach: 4
Environment: 1

Overall Impact: The application is a resubmission of an R34 which seeks to adapt the evidence based mentor mother intervention for pregnant and lactating (PLP) who use alcohol in South Africa and may be at increased risk for HIV acquisition or poor HIV outcomes for themselves or their children. The proposal is significant given alcohol use is quite prevalent in South Africa among PLP and can cause fetal alcohol syndrome, poor birth outcomes and child health outcomes in addition to increase risk for vertical transmission and poor treatment outcomes for mothers and children. The intervention will be status neutral and link participants to HIV Tx or PrEP as needed. MM has been shown to improved ANC and HIV outcomes among women living with HIV in South Africa but not yet addressed the link with alcohol use. The primary outcome for the R34 is alcohol use reduction to be achieved through a multi module intervention to be implemented over 6 months which includes peer based counseling sessions using motivational interviewing to address risks and provide supports related to harmful drinking and promote HIV risk reduction, PrEP and ART as relevant. Aim 1 is a mixed method study of perceptions of alcohol during pregnancy to inform the model, Aim 2 focuses on adapting the MM model (ADAPTT IT) and both engage PLP, providers and community leaders; Aim 3 examines acceptability and feasibility of the adapted model (n=100).

1. Significance:

Strengths

- Alcohol use including among PLP is prevalent and has been known to impact HIV outcomes both in terms of prevention and care; including in high burden HIV settings such as South Africa which has one of the highest burden of HIV among women in the world.

Weaknesses

- None noted by reviewer.

2. Investigator(s):

Strengths

- MPI: Joseph-Davey (UCLA) is the contact PI an epidemiologist and associate professor in school of medicine; she has significant experience in RSA and is a seasoned researcher of HIV among women and children and other vulnerable groups.
- MPI: Essack is a social scientist and chief research specialist (HSRC) versed in the study of psychosocial factors impacting HIV outcomes in RSA using community-based approaches.
- The MPIs complement each other and have a strong communication, collaboration and conflict management plan. They will collaborate with team members Belin (stats) who is a professor at UCLA, research specialist Peterson at HSRC and postdoc Miller at SDSU who are both focused on alcohol research in SSA. Also at HSRC is Heidi van Rooyen who has previously used the mentor mothers model in RSA.

Weaknesses

- It is a relatively large team for an R34 however roles are fairly well delineated and plans contemplate capacity building within the study for more junior investigators in both sites.

3. Innovation:

Strengths

- Adaptation of MM to engage PLP who use alcohol is innovative as is doing so using a status neutral approach.

Weaknesses

- Methods are not particularly innovative (IDIs, FGDs, etc.)

4. Approach:

Strengths

- Aims to developed evidence based intervention for reducing negative influence of alcohol among PLP and in turn improve HIV outcomes for mothers and children.
- Use of ADAPT-ITT using mixed methods approach and community engagement.
- Implementation indicators as well as biomarker outcomes (status neutral including biomarkers for alcohol, PrEP and ART).
- Strong preliminary work by team in the study population and site.

Weaknesses

- Outcome alcohol reduction but to power larger trial impact on secondary outcomes; Small numbers for HIV – and HIV+ PLP in terms of the secondary outcomes.
- Limited detail on implementation outcome measurement (feasibility/acceptability).
- Analysis plan doesn't contemplate causal pathway model proposed in much detail.

5. Environment:

Strengths

- UCLA, HSRC, SDSU all have excellent environments to support the investigators and proposed study aims.

Weaknesses

- None noted by reviewer.

Study Timeline:

Strengths

- Well developed timeline contemplating recruitment, fieldwork and analysis.

Weaknesses

- None noted by reviewer.

Protections for Human Subjects:

Acceptable Risks and/or Adequate Protections

- No concerns

Inclusion Plans:

- Sex/Gender: Distribution justified scientifically
- Race/Ethnicity: Distribution justified scientifically
- Inclusion/Exclusion Based on Age: Distribution justified scientifically
- Inclusion of PLP is justified as are stakeholders eligibility criteria

Vertebrate Animals:

Not Applicable (No Vertebrate Animals)

Biohazards:

Not Applicable (No Biohazards)

Resubmission:

- Responsive to prior critiques around prior literature on MM as well as addition of key team members including a statistician and more senior faculty in HRSC in RSA; most of the other critiques involved lack of detail on the methods which have been addressed

Applications from Foreign Organizations:
Justified

- RSA justified site for the MM+ model given prevalence of alcohol use and hIV

Resource Sharing Plans:
Acceptable

- Well designed dissemination plans and resource sharing

Budget and Period of Support:
Recommend as Requested

CRITIQUE 2

Significance: 2
Investigator(s): 1
Innovation: 2
Approach: 5
Environment: 1

Overall Impact: South Africa has the highest combined prevalence of alcohol use in pregnancy and HIV in the world. Alcohol use and HIV prevention and treatment are interrelated. The proposed research seeks to develop an evidence-based, community-informed clinic-based intervention to minimize negative health outcomes due to alcohol and HIV in pregnancy—the first of its kind. If found effective, the proposed clinic-based intervention could impact maternal and child health through (1) reduction of alcohol use during pregnancy and breastfeeding, (2) reduction of HIV risk among HIV negative pregnant women, and (3) improved HIV treatment outcomes among pregnant women living with HIV. Overall, the proposed methodology and analyses are well-reasoned and appropriate to accomplish the specific aims of the project. Further, the researchers collectively have the necessary training and previous experience, and the appropriate institutional environment, to successfully implement the proposed approach. The researchers propose adapting and refining an evidenced-based HIV prevention intervention called Mentor Mothers (MM), to address perinatal alcohol use and HIV using mixed methods and community-based action research, and then pilot testing the intervention in a RCT to assess its feasibility and acceptability. They plan to use innovative methods for delivering biofeedback counseling on alcohol use, PrEP and ART adherence to participants, and plan to measure biological outcomes in the RCT. If the aims are achieved, the study findings will likely impact public health practice through improved community awareness of perinatal alcohol use and HIV, and the adaptation of an evidence-based, scalable intervention which can be further refined, scaled up and ultimately integrated into routine service delivery in South Africa, and possibly similar contexts in Africa. The likelihood of the project exerting a sustained and powerful influence depends on the scalability of the adapted intervention. The MM intervention has been shown to be effective at improving HIV and antenatal care outcomes, contributing to the prevention of vertical HIV transmission in South Africa and in other countries. Further, interventions based on the MM model have been adapted and scaled widely across Africa. However, the efficacy and cost-effectiveness of the proposed adapted MM intervention is

unknown. Further, it does not appear that the researchers are planning to track the implementation costs which greatly affects scalability and should be considered even during the pilot phase.

1. Significance:

Strengths

- South Africa has high antenatal HIV prevalence (31%) and pediatric HIV incidence >750/100,000 live births. Further, there is an urgent need to reduce perinatal alcohol use in the proposed study area in Western Cape which has the highest rates of fetal alcohol syndrome in the world.
- Alcohol use and HIV prevention and treatment are interrelated. Therefore, intervening on both public health problems in the proposed study area, has potential to positively impact the health of mothers, child, and families.
- The researchers plan to adapt an evidenced-based HIV prevention intervention to address alcohol use and HIV risk during pregnancy. The intervention, called Mentor Mothers (MM), has been shown to be effective at improving HIV and antenatal (ANC) care outcomes, contributing to the prevention of vertical HIV transmission in South Africa and in other countries. A recent cluster RCT of home-based delivery of MM in South Africa demonstrated reduced alcohol use in pregnant and postpartum women.
- Over the last 20 years interventions based on the MM model have been adapted and scaled across 10 African countries.
- The research team's recently completed Pilot RCT in Cape Town suggests that provision of PrEP adherence biofeedback improves adherence among postpartum women. Further, personalized feedback based on self-reported alcohol use has shown promise in reducing use. The researchers plan to incorporate recent alcohol use, PrEP, and ART adherence biofeedback into the adapted intervention using quick, accurate, and low-cost urine tests.
- If the aims are achieved, the study findings will impact public health practice through (1) improved community awareness of perinatal alcohol use and HIV, and (2) the adaptation of an evidence-based, scalable intervention to address alcohol use and HIV risk during pregnancy which can be further refined, scaled up and ultimately integrated into routine service delivery in South Africa, and possibly similar contexts in Africa.

Weaknesses

- Policymakers will want to know how much the intervention will cost before making decisions to adopt and scale it. It does not appear that the researchers are planning to track the implementation costs. While I recognize the proposed research is pilot testing an intervention, implementation costs could still be documented to help inform future decisions about the intervention.
- Related, while the researchers note that the MM model has been used to implement cost-effective interventions to address HIV risk in other contexts, they may find that adding activities to address perinatal alcohol use to the MM intervention requires more resources than policymakers are willing/able to spend, and this could limit uptake of the adapted intervention. It is not clear if the research team will set parameters for the types of adaptions and activities that they will implement to contain the intervention's costs to preserve its scalability.

2. Investigator(s):

Strengths

- The leadership approach and organization structure are clear and appropriate given the proposed roles and responsibilities for each team member.
- The proposed team has been collaborating to build an evidence base demonstrating the need for a culturally tailored intervention to address alcohol use and HIV among pregnant women in South Africa.
- The proposed investigators have the training and previous experiences to implement the proposed approach to all three aims.
- Joseph Davey, PI, based in Cape Town, has conducted research among pregnant and lactating people (PLP), including a large cohort study of over 1300 PLP on PrEP in Cape Town, and has expertise in epidemiologic methods, implementation science studies and clinical trials in South Africa.
- Essack, MPI, psychologist and social scientist based in Cape Town, expertise in socio-behavioral research, will oversee development of Aims 1 and 2.
- Petersen, Co-I, researcher, with expertise in substance use, IPV, and community-based participatory action research (CBPAR), responsible for implementation of study with FARR.
- Belin, Co-I, Biostatistician, will develop statistical analysis plan and conduct statistical analyses.
- Miller, site PI and co-I, expertise in qualitative data collection and analysis, HIV epidemiology and substance use, will lead the mixed methods analysis for Aim 1 and qualitative analysis for Aim 2.
- van Rooyen, Co-I, expertise in clinical psychologist and HIV prevention and treatment, including counseling, testing, and community-based approaches.
- Hahn (Consultant) is an expert on PEth measurement.

Weaknesses

- Miller is proposed to be the site PI however, that does not seem practical since Miller is based in San Diego. However, in another of the application Essack is referred to as the site PI which makes more sense since Essack is based in Cape Town.
- The description of roles for some team members seems to vary in the different parts of the application.

3. Innovation:

Strengths

- The proposed research plans to adapt an effective HIV prevention intervention to include perinatal alcohol use, and if found successful, this would be the first evidence-based, community-informed intervention to minimize negative health outcomes due to alcohol and HIV in pregnancy.
- This would be one of the first studies to use PEth as an objective measure of alcohol use among pregnant and lactating people (PLP) in a high HIV and alcohol using community. This is important advancement in measurement because previous research has identified biological factors and race/ethnicity differences in PEth sensitivity.

Weaknesses

- None noted by reviewer.

4. Approach:

Strengths

- Adaption of an evidence-based intervention that has been shown successful in South Africa and other countries, and has been widely adopted in Africa, increases chances that the new intervention will be effective and scalable.
- Proposes adapting the intervention using mixed methods and community-based action research, and refining the intervention with potential beneficiaries, providers, and community leaders before evaluating. The intensive and iterative involvement of the community is likely to increase the intervention's feasibility and acceptability.
- Plans to evaluate feasibly and acceptably of the adapted intervention using a rigorous method (RCT).
- Objective biological outcomes for RCT: reduced alcohol use at 6m using PEth is primary outcome and PrEP and ART continued use at 6m via urine tenofovir levels at 6m are secondary outcomes.
- Use of an enhanced HIV attention control is a best practice.
- Use of different staff and spaces to implement the intervention within the ANC clinic to reduce contamination.
- Will assess any differences between RCT participants and refusers.
- Strong measures of intervention fidelity include recording 20% of the sessions and using a checklist.
- Conducting interviews to assess organizational, providers, and PLP's views on acceptability, feasibility, and scalability of implementation of intervention.
- The explanatory sequential mixed methods design in Aim 3 (where the analysis of the quantitative data will inform the purposively selected samples of participants for the interviews) will allow explanation of emergent and unexpected themes regarding barriers to intervention implementation, uptake, and fidelity.
- Data from participants and healthcare providers will be triangulated to provide a holistic understanding of acceptability of the intervention.
- Strong retention plan in place for all study arms.
- Study has a plan to actively detect and treat trauma that the mentor mothers may experience because of implementing the intervention.
- Plans to strengthen research capacity of local organization, Foundation for Alcohol Related Research (FARR).

Weaknesses

- It appears that the intervention will only be implemented in one ANC clinic. This means that the researchers will not be able to learn how clinic-level factors may influence feasibility, acceptability, fidelity of the intervention.
- While the researchers provide a citation justifying only conducting one FGD with healthcare providers and one FGD with community leaders for Aim 1, it seems risky to plan for only one group because a dominant participant and/or a power dynamic between participants might derail the group. Moreover, having at least two FGDs with each study population would allow the researchers to check if similar salient themes are arising across the groups.

- It is unclear if the participants in Aim 1 will be allowed to participate in the activities for Aim 2.
- It is not clear whether the providers (midwives, nurses, and counselors) who will implement the interventions in the intervention and control clinics will be hired by the study or if they will be existing staff from the public ANC clinic (i.e., Department of Health employees).
- While I understand why the mentor mothers will not provide counseling to the control group, it is not clear why the services in the control arm will be provided by HIV counselors whereas the services in the intervention arm will be provide by a nurse. It may be possible the different cadres of healthcare workers approach HIV counseling differently and this may bias the study results.
- Related to the above point, under the section describing the MM model, the researchers note that the MM intervention does not provide incentives and suggests that the Department of Health could have their community health workers deliver the intervention as part of the standard of care. However, community health workers are not the implementers of the adapted intervention in the proposed research. If the researchers and government intend for community health workers to ultimately implement the intervention than the pilot should also use this cadre to implement the intervention.
- It is unclear if the mentor mothers will be paid for their work.
- The researchers mention that ART adherence will be evaluated in conjunction with viral loads, but it is not clear how the researchers will obtain viral loads and what they will do with the viral load data. The Data and Safety Monitoring Plan briefly mentions that electronic medical record data will be extracted, but there are no further details on who will extract these data, how they will be transmitted to the researchers and how they will be used in the study.
- It is unclear why pregnancy participants under 18 years of age will not be enrolled in the RCT.
- In the recruitment and retention plan the details about how the pregnant women are first approached about the study are lacking. For example, is it routine for all women who attend ANC at the clinic to be asked to complete an assessment measuring alcohol use, and then ANC providers examine the score on this assessment and counsel based on the results? During the study enrollment period, will the ANC providers tell the women who qualify based on their scores about the study? Where and when does this happen? If the women are interested in the study, how do they reach the trained study staff member to confirm eligibility and go through the informed consent process?
- It is not clear what factors, characteristics, etc. will be considered when purposively selecting the partners, drinking peers, healthcare providers, and community leaders for the proposed research. It is also unclear who will make the selection of these potential participants and how the potential participants will be approached to first learn about the study (as described in the bullet above).

5. Environment:

Strengths

- The principal investigator’s institution, UCLA, along with the other investigators’ institutions, are appropriate to support the investigators on this research. Specifically, they have the space and equipment to enable the investigators to develop the study protocol and data collection instruments, store the data, and conduct the proposed data analyses.
- Human Sciences Research Council (HSRC) has conducted surveys across South Africa for the past 20 years including those on HIV, IPV and in vulnerable populations.

- FARR to conduct community entry, recruitment, and data collection.

Weaknesses

- None noted by reviewer.

Study Timeline:

Strengths

- The time allocated to most of the proposed activities appears appropriate. The exception is the time allocated to analyze and disseminate the pilot study results does not seem long enough in relation to when those data will be collected.

Weaknesses

- The time allocated for Aim 1 seems long given the limited data that are proposed to be collected for that aim compared to the short amount of time allocated to data analysis and dissemination for Aim 3 when there are a lot of data proposed to be collected.

Protections for Human Subjects:

Acceptable Risks and/or Adequate Protections

- The research team identified the risks that may arise in implementing their proposed research and they described appropriate plans to reduce these risks.

Data and Safety Monitoring Plan (Applicable for Clinical Trials Only):

Acceptable

- o The plan appears appropriate for this study.

Inclusion Plans:

- Sex/Gender: Distribution justified scientifically
- Race/Ethnicity: Distribution justified scientifically
- Inclusion/Exclusion Based on Age: Distribution not justified scientifically
- It is unclear why pregnancy participants under 18 years of age will not be enrolled in the RCT.

Vertebrate Animals:

Not Applicable (No Vertebrate Animals)

Biohazards:

Not Applicable (No Biohazards)

Resubmission:

- Responsive to previous comments.

Applications from Foreign Organizations:

Justified

- The proposed research takes place in South Africa and requires collaboration with organizations in South Africa. The proposed research could not be conducted in the US or any other setting.

Budget and Period of Support:

Recommend as Requested

CRITIQUE 3

Significance: 2
Investigator(s): 1
Innovation: 2
Approach: 6
Environment: 1

Overall Impact: The proposed study seeks to use the ADAPT-ITT methodology to adapt the Mentor Mothers (MM) intervention for substance use among pregnant and lactating persons (PLP) to increase PrEP initiation and encourage HIV adherence among seropositive women. Building upon formative work by the investigative team, Dr Joseph Davey and Dr. Essack propose a community-engaged approach of inquiry of determinants of alcohol use among pregnant women (IDIs of intimate partners and drinking partners; FGDs of healthcare workers and community leaders) to inform intervention adaptation. In the next phase, the investigators seek to determine a Theory of Change for intervention adaptation through community stakeholder workshops. The final part of the proposed study is a pilot intervention trial randomizing 100 pregnant mothers who have admitted to alcohol use in the six weeks prior to enrollment to standard of care v. the MM+ intervention supported by urine biomarker informed (urine TFV and urine alcohol metabolites) approach to counseling, ultimately measuring alcohol use reduction as a primary outcome. This proposal addresses a consequential syndemic of alcohol use and HIV, and the investigators attempt to bring in evidence based practice for alcohol use into the HIV space is commendable and novel. The use of urine biomarkers add another level of validation to the study's output. The investigators have complementary expertise and have an emerging record of collaboration. Unfortunately, there are fundamental issues with the approach (ie the lack of definition of the study population by serostatus, lack of clarity of trial design and intervention sequence, the attempt of the study to address treatment and prevention simultaneously, and questions of feasibility of recruitment in the reported timeframe) that impact my overall assessment of the proposal.

1. Significance:

Strengths

- The proposed work addresses an important syndemic: alcohol use and HIV among pregnant women.
- Bringing best practices in substance use reduction for the mitigation of HIV risk among pregnant women in SA is novel and highly significant. If successful, the yield of this work could result in a new paradigm for addressing HIV risk in this key population.
- Although many investigators have mobilized multi-level community input to address alcohol use in SA previously, the investigators' intent to engage multiple community stakeholders and social networks in this broader conversation pertaining to why alcohol use in pregnant use is prevalent

in Western Cape is highly significant. This formative work has the potential to inform multiple future studies.

Weaknesses

- The study's attempt to address alcohol use reduction, optimizing PrEP utilization and HIV treatment adherence is problematic. It feels like the study is trying to answer too many questions at the same time. A focus on just HIV prevention or ART adherence would be well advised.

2. Investigator(s):

Strengths

- The justification for an MPI format is well founded. Dr. Joseph-Davey brings significant experience in epidemiology and implementation science methodology among PLP in South Africa, particularly in the realm of HIV. Dr. Essack brings experience in community-based participatory research among marginalized women in South Africa. Their skills are complementary, and the study team organization is well presented.
- Dr. Miller will bring expertise in qualitative methods to the group and has collaborated with the UCLA team in the past.
- Dr. Petersen brings clinical expertise and local expertise from the Saldanha Bay area.

Weaknesses

- None noted by reviewer.

3. Innovation:

Strengths

- The investigators use of urine biomarkers with participant feedback for the reduction of alcohol use is innovative.
- Bring MM+ an established evidence-based intervention for pregnant mothers with HIV to address alcohol use in a status-neutral manner is highly innovative.

Weaknesses

- It is really peculiar that the only innovation stated by the investigators of the proposed work is the use of urine biomarkers. Nothing was mentioned about the actual adapted MM+ intervention to address alcohol use, the main driver of innovation for this study.

4. Approach:

Strengths

- The use of ADAPT-ITT to adapt the MM framework to HIV treatment and prevention is very detailed and well mapped.
- The incorporation of intimate partners and drinking partners of PLP as well as healthcare providers and community leaders is very thoughtful and makes for a robust multilevel assessment of the problem of alcohol use among PLP.
- The use of a Theory of Change approach is a strength of this proposal and allows for the values of the co-adaptation process to be imprinted on the final MM+ strategy.

Weaknesses

- The eligible population is not well defined. It is unclear what proportion of seronegative and seropositive persons the investigators are expecting to recruit in each arm. Whether HIV screening will be conducted at study enrollment and how that result may impact the effect of the intervention is also not addressed.
- More detail could have been presented about how mentor mothers were going to be selected from the community.
- It is unclear why only women in the intervention group will received their enhanced counseling from a trained study nurse. Whether study nurse counseling was part of the proposed MM+ intervention was not addressed.
- Figure 3 is confusing. It mentions urine tenofovir testing and urine EtG testing, but I would imagine that pathways through the trial would be slightly different for HIV seronegative and seropositive patients.
- Details on the power calculation seem to be sparse. The determination of an effect size would have been adequate at this point given that this adaptation is still in the pilot phase.
- Unclear how feasible it is to recruit 100 PLP who use alcohol in 6 months at a single facility.
- The rationale for the power calculation seems sparse, and I would imagine at this stage that obtaining a general range of the effect size of the intervention would be most important. There is also some discrepancy between the power calculation described in the text and the human subject section.
- The sample sizes for the secondary outcomes will be small. How data will be analyzed for subset of persons with HIV (adherence outcome) v. subset of persons without HIV is not described.

5. Environment:

Strengths

- UCLA has an established track record of supporting the development of population level interventions for the prevention and treatment of HIV.
- The HSRC is an excellent partner for the investigators and is well-positioned to see through the execution of the research strategy as planned.

Weaknesses

- No concerns identified.

Study Timeline:

Strengths

- The timeline has adequate detail

Weaknesses

- Six months to recruit 100 pregnant mothers who admit to alcohol use is not a lot of time.

Protections for Human Subjects:

Acceptable Risks and/or Adequate Protections

- More detail should be given on how intimate partners and drinking partners of these mothers will be identified for interview, and measures to protect identity of prospective participants in this process.

Data and Safety Monitoring Plan (Applicable for Clinical Trials Only):

Acceptable

Inclusion Plans:

- Sex/Gender: Distribution justified scientifically
- Race/Ethnicity: Distribution justified scientifically
- For NIH-Defined Phase III trials, Plans for valid design and analysis: Scientifically acceptable
- Inclusion/Exclusion Based on Age: Distribution justified scientifically

Vertebrate Animals:

Not Applicable (No Vertebrate Animals)

Biohazards:

Not Applicable (No Biohazards)

Resubmission:

- Prior critiques are adequately addressed

Applications from Foreign Organizations:

Justified

- Justification for the involvement of HSRC Cape Town in this work is well delineated

Resource Sharing Plans:

Acceptable

Budget and Period of Support:

Recommend as Requested

THE FOLLOWING SECTIONS WERE PREPARED BY THE SCIENTIFIC REVIEW OFFICER TO SUMMARIZE THE OUTCOME OF DISCUSSIONS OF THE REVIEW COMMITTEE, OR REVIEWERS' WRITTEN CRITIQUES, ON THE FOLLOWING ISSUES:

PROTECTION OF HUMAN SUBJECTS: ACCEPTABLE

INCLUSION OF WOMEN PLAN: ACCEPTABLE

INCLUSION OF MINORITIES PLAN: ACCEPTABLE

INCLUSION ACROSS THE LIFESPAN: UNACCEPTABLE
The panel expressed the rationale for excluding PLP under the age 18 was not clear.

COMMITTEE BUDGET RECOMMENDATIONS: The budget was recommended as requested.

Footnotes for 1R34AA030942-01A1; PI Name: JOSEPH DAVEY, DVORA

NIH has modified its policy regarding the receipt of resubmissions (amended applications).See Guide Notice NOT-OD-18-197 at https://grants.nih.gov/grants/guide/notice-files/NOT-OD-18-197.html. The impact/priority score is calculated after discussion of an application by averaging the overall scores (1-9) given by all voting reviewers on the committee and multiplying by 10. The criterion scores are submitted prior to the meeting by the individual reviewers assigned to an application, and are not discussed specifically at the review meeting or calculated into the overall impact score. Some applications also receive a percentile ranking. For details on the review process, see http://grants.nih.gov/grants/peer_review_process.htm#scoring.

MEETING ROSTER

Population and Public Health Approaches to HIV/AIDS Study Section
Healthcare Delivery and Methodologies Integrated Review Group
CENTER FOR SCIENTIFIC REVIEW
PPAH
11/15/2023 - 11/16/2023

Notice of NIH Policy to All Applicants: Meeting rosters are provided for information purposes only. Applicant investigators and institutional officials must not communicate directly with study section members about an application before or after the review. Failure to observe this policy will create a serious breach of integrity in the peer review process, and may lead to actions outlined in NOT-OD-22-044 at https://grants.nih.gov/grants/guide/notice-files/NOT-OD-22-044.html, including removal of the application from immediate review.

CHAIRPERSON(S)

YOUNG, APRIL M, PHD
PROFESSOR
DEPARTMENT OF EPIDEMIOLOGY AND
ENVIRONMENTAL HEALTH
COLLEGE OF PUBLIC HEALTH
UNIVERSITY OF KENTUCKY
LEXINGTON, KY 40536

MEMBERS

ABUOGI, LISA L, MD
ASSOCIATE PROFESSOR
DEPARTMENT OF PEDIATRICS
SCHOOL OF MEDICINE
UNIVERSITY OF COLORADO, DENVER
AURORA, CO 80045

AMIRKHANIAN, YURI A, PHD
PROFESSOR
DEPARTMENT OF PSYCHIATRY AND BEHAVIORAL MEDICINE
CENTER FOR AIDS INTERVENTION RESEARCH
MEDICAL COLLEGE OF WISCONSIN
MILWAUKEE, WI 53202

BALASUBRAMANIAN, RAJI, DSC
ASSOCIATE PROFESSOR
DEPARTMENT OF BIOSTATISTICS AND EPIDEMIOLOGY
SCHOOL OF PUBLIC HEALTH AND HEALTH SCIENCES
UNIVERSITY OF MASSACHUSETTS
AMHERST, MA 01003

BARNIGHAUSEN, TILL, MD, SCD
PROFESSOR AND DIRECTOR
HEIDELBERG INSTITUTE OF GLOBAL HEALTH
UNIVERSITY OF HEIDELBERG
HEIDLEBERG, GERMANY 69120
GERMANY

BENDAVID, ERAN, MD
ASSOCIATE PROFESSOR
DEPARTMENT OF MEDICINE
STANFORD UNIVERSITY
STANFORD, CA 94305

BIRKETT, MICHELLE, PHD
ASSOCIATE PROFESSOR
DEPARTMENT OF MEDICAL SOCIAL SCIENCES
AND PREVENTIVE MEDICINE
FEINBERG SCHOOL OF MEDICINE
NORTHWESTERN UNIVERSITY
CHICAGO, IL 60611

BURKE, HOLLY MCCLAIN, PHD *
SCIENTIST
REPRODUCTIVE, MATERNAL, NEWBORN, AND
CHILD HEALTH DIVISION
FHI 360
DURHAM, NC 27701

CHRISTOPOULOS, KATERINA A, MD
PROFESSOR OF MEDICINE
HIV/AIDS DIVISION
SAN FRANCISCO GENERAL HOSPITAL
UNIVERSITY OF CALIFORNIA, SAN FRANCISCO
SAN FRANCISCO, CA 94110

DARBES, LYNAE A, PHD
ASSOCIATE PROFESSOR
DEPARTMENT OF HEALTH BEHAVIOR
AND BIOLOGICAL SCIENCES
SCHOOL OF NURSING
UNIVERSITY OF MICHIGAN
ANN ARBOR, MI 48109

FERGUSON, TEKEDA F, PHD *
ASSOCIATE PROFESSOR
DEPARTMENT OF EPIDEMIOLOGY
SCHOOL OF PUBLIC HEALTH
LOUISIANA STATE UNIVERSITY HEALTH SCIENCES CENTER
NEW ORLEANS, LA 70112

FERKET, BART, MD, PHD
ASSOCIATE PROFESSOR
DEPARTMENT OF POPULATION HEALTH SCIENCE AND POLICY
ICAHN SCHOOL OF MEDICINE AT MOUNT SINAI
NEW YORK, NY 10029

GENG, ELVIN H, MD *
PROFESSOR
DEPARTMENT OF INTERNAL MEDICINE
DIVISION OF INFECTIOUS DISEASES
SCHOOL OF MEDICINE
WASHINGTON UNIVERSITY
ST. LOUIS, MO 63110

GOLIN, CAROL E, MD
PROFESSOR
DEPARTMENT OF MEDICINE, DIVISION OF GENERAL MEDICINE AND CLINICAL EPIDEMIOLOGY
DEPARTMENT OF HEALTH BEHAVIOR
UNIVERSITY OF NORTH CAROLINA
CHAPEL HILL, NC 27599

HAIDER, MOHAMMAD RIFAT, PHD *
ASSISTANT PROFESSOR
DEPARTMENT OF HEALTH POLICY AND MANAGEMENT
COLLEGE OF PUBLIC HEALTH
UNIVERSITY OF GEORGIA
ATHENS, GA 30602

HEFFRON, RENEE A, PHD *
PROFESSOR AND DIRECTOR
DEPARTMENT OF MEDICINE
CENTER FOR AIDS RESEARCH
UNIVERSITY OF ALABAMA AT BIRMINGHAM
BIRMINGHAM, AL 35233

HOGAN, JOSEPH W, SCD
PROFESSOR AND CHAIR
DEPARTMENT OF BIOSTATISTICS
SCHOOL OF PUBLIC HEALTH
BROWN UNIVERSITY
PROVIDENCE, RI 02912

JENNESS, SAMUEL, PHD
ASSOCIATE PROFESSOR
DEPARTMENT OF EPIDEMIOLOGY
ROLLINS SCHOOL OF PUBLIC HEALTH
EMORY UNIVERSITY
ATLANTA, GA 30322

KERRIGAN, DEANNA L, PHD
PROFESSOR AND CHAIR
DEPARTMENT OF PREVENTION AND COMMUNITY HEALTH
MILKEN INSTITUTE SCHOOL OF PUBLIC HEALTH
GEORGE WASHINGTON UNIVERSITY
WASHINGTON, DC 20052

LANCASTER, KATHRYN ELIZABETH, PHD *
ASSOCIATE PROFESSOR
WAKE FOREST UNIVERSITY
WINSTON-SALEM, NC 27101

LAU, BRYAN, PHD
PROFESSOR
DEPARTMENT OF EPIDEMIOLOGY
BLOOMBERG SCHOOL OF PUBLIC HEALTH
JOHNS HOPKINS UNIVERSITY SCHOOL OF MEDICINE
BALTIMORE, MD 21287

LELUTIU-WEINBERGER, CORINA, PHD
ASSOCIATE PROFESSOR
SCHOOL OF NURSING
COLUMBIA UNIVERSITY
NEW YORK, NY 10032

LEWIS, CRYSTAL FULLER, PHD *
PROFESSOR
DEPARTMENT OF PSYCHIATRY
GROSSMAN SCHOOL OF MEDICINE
NEW YORK UNIVERSITY
NEW YORK, NY 10016

LIMA, VIVIANE DIAS, SCD *
ASSOCIATE PROFESSOR
DEPARTMENT OF MEDICINE
DIVISION OF INFECTIOUS DISEASE
UNIVERSITY OF BRITISH COLUMBIA
VANCOUVER, BC V6Z1Y6
CANADA

MCCLELLAND, RAYMOND S, MD
PROFESSOR
DEPARTMENTS OF MEDICINE, EPIDEMIOLOGY, AND GLOBAL HEALTH
SCHOOL OF MEDICINE
UNIVERSITY OF WASHINGTON
SEATTLE, WA 98104

NAPIERALA, SUE, PHD *
RESEARCH EPIDEMIOLOGIST
RESEARCH TRIANGLE INSTITUTE
BERKELEY, CA 94704

NIJHAWAN, ANK E, MD
ASSOCIATE PROFESSOR
DIVISION OF INFECTIOUS DISEASES
UNIVERSITY OF TEXAS SOUTHWESTERN MEDICAL CENTER
DALLAS, TX 75390

OKEKE, NWORA LANCE, MD *
ASSOCIATE PROFESSOR OF MEDICINE AND POPULATION HEALTH SCIENCES
DEPARTMENT OF MEDICINE
SCHOOL OF MEDICINE
DUKE UNIVERSITY
DURHAM, NC 27710

OUTLAW, ANGULIQUE Y, PHD
ASSOCIATE PROFESSOR
DEPARTMENT OF FAMILY MEDICINE AND
PUBLIC HEALTH SCIENCES
SCHOOL OF MEDICINE
WAYNE STATE UNIVERSITY
DETROIT, MI 48201

PHO, MAI T, MD
ASSOCIATE PROFESSOR
DEPARTMENT OF MEDICINE
SECTION OF INFECTIOUS DISEASES AND GLOBAL HEALTH
UNIVERSITY OF CHICAGO MEDICAL CENTER
CHICAGO, IL 60637

RAMIREZ (KITCHEN), CHRISTINA MICHELLE, PHD *
PROFESSOR
DEPARTMENT OF BIOSTATISTICS
SCHOOL OF PUBLIC HEALTH
UNIVERSITY OF CALIFORNIA, LOS ANGELES
LOS ANGELES, CA 90095

WESTERGAARD, RYAN PATRICK, MD, PHD *
ASSOCIATE PROFESSOR
DEPARTMENT OF MEDICINE
SCHOOL OF MEDICINE AND PUBLIC HEALTH
UNIVERSITY OF WISCONSIN
MADISON, WI 53705

WONG, FRANK Y, PHD *
MCKENZIE ENDOWED PROFESSOR OF HEALTH EQUITY
RESEARCH
CENTER OF POPULATION SCIENCES FOR HEALTH EQUITY
COLLEGE OF SOCIAL WORK
FLORIDA STATE UNIVERSITY
TALLAHASSEE, FL 32310

ZHANG, CHEN, PHD *
ASSISTANT PROFESSOR
SCHOOL OF NURSING
UNIVERSITY OF ROCHESTER
ROCHESTER, NY 14620

MAIL REVIEWER(S)

ENNS, EVA A, PHD
ASSOCIATE PROFESSOR
DIVISION OF HEALTH POLICY AND MANAGEMENT
SCHOOL OF PUBLIC HEALTH
UNIVERSITY OF MINNESOTA
MINNEAPOLIS, MN 55455

SCIENTIFIC REVIEW OFFICER

MADKOUR, AUBREY SPRIGGS, PHD
SCIENTIFIC REVIEW OFFICER
CENTER FOR SCIENTIFIC REVIEW
NATIONAL INSTITUTES OF HEALTH
BETHESDA, MD 20892

EXTRAMURAL SUPPORT ASSISTANT

SMITH, ALETHIA ALEXANDREA
EXTRAMURAL SUPPORT ASSISTANT
THE CENTER FOR SCIENTIFIC REVIEW
THE NATIONAL INSTITUTES OF HEALTH
BETHESDA, MD 20892

OTHER REVIEW STAFF

MCREE, ANNIE LAURIE, PHD
SCIENTIFIC REVIEW OFFICER
CENTER FOR SCIENTIFIC REVIEW
NATIONAL INSTITUTES OF HEALTH
BETHESDA, MD 20892

* Temporary Member. For grant applications, temporary members may participate in the entire meeting or may review only selected applications as needed.

Consultants are required to absent themselves from the room during the review of any application if their presence would constitute or appear to constitute a conflict of interest.
